# Untreated clinical course of cerebral cavernous malformations: a prospective, population-based cohort study

**DOI:** 10.1016/S1474-4422(12)70004-2

**Published:** 2012-03

**Authors:** Rustam Al-Shahi Salman, Julie M Hall, Margaret A Horne, Fiona Moultrie, Colin B Josephson, Jo J Bhattacharya, Carl E Counsell, Gordon D Murray, Vakis Papanastassiou, Vaughn Ritchie, Richard C Roberts, Robin J Sellar, Charles P Warlow, for the Scottish Audit of Intracranial Vascular Malformations (SAIVMs) collaborators

**Affiliations:** a Division of Clinical Neurosciences, Centre for Clinical Brain Sciences, University of Edinburgh, Edinburgh, UK; b Centre for Population Health Sciences, University of Edinburgh, Edinburgh, UK; c Radiology Department, Royal Victoria Infirmary, Newcastle-upon-Tyne, UK; d Institute of Neurological Sciences, Southern General Hospital, Glasgow, UK; e Division of Applied Health Sciences, University of Aberdeen, Aberdeen, UK; f Fauldhouse Health Centre, Fauldhouse, UK; g Neurology Department, Ninewells Hospital and Medical School, Dundee, UK

## Abstract

**Background:**

Cerebral cavernous malformations (CCMs) are prone to bleeding but the risk of intracranial haemorrhage and focal neurological deficits, and the factors that might predict their occurrence, are unclear. We aimed to quantify these risks and investigate whether they are affected by sex and CCM location.

**Methods:**

We undertook a population-based study using multiple overlapping sources of case ascertainment (including a Scotland-wide collaboration of neurologists, neurosurgeons, stroke physicians, radiologists, and pathologists, as well as searches of registers of hospital discharges and death certificates) to identify definite CCM diagnoses first made in Scottish residents between 1999 and 2003, which study neuroradiologists independently validated. We used multiple sources of prospective follow-up both to identify outcome events (which were assessed by use of brain imaging, by investigators masked to potential predictive factors) and to assess adults' dependence. The primary outcome was a composite of intracranial haemorrhage or focal neurological deficits (not including epileptic seizure) that were definitely or possibly related to CCM.

**Findings:**

139 adults had at least one definite CCM and 134 were alive at initial presentation. During 1177 person-years of follow-up (completeness 97%), for intracranial haemorrhage alone the 5-year risk of a first haemorrhage was lower than the risk of recurrent haemorrhage (2·4%, 95% CI 0·0–5·7 *vs* 29·5%, 4·1–55·0; p<0·0001). For the primary outcome, the 5-year risk of a first event was lower than the risk of recurrence (9·3%, 3·1–15·4 *vs* 42·4%, 26·8–58·0; p<0·0001). The annual risk of recurrence of the primary outcome declined from 19·8% (95% CI 6·1–33·4) in year 1 to 5·0% (0·0–14·8) in year 5 and was higher for women than men (p=0·01) but not for adults with brainstem CCMs versus CCMs in other locations (p=0·17).

**Interpretation:**

The risk of recurrent intracranial haemorrhage or focal neurological deficit from a CCM is greater than the risk of a first event, is greater for women than for men, and declines over 5 years. This information can be used in clinical practice, but further work is needed to quantify risks precisely in the long term and to understand why women are at greater risk of recurrence than men.

**Funding:**

UK Medical Research Council, Chief Scientist Office of the Scottish Government, and UK Stroke Association.

## Introduction

Cerebral cavernous malformations (CCMs) are common, occurring in one in about 600 neurologically asymptomatic people as evident on MRI scans and one in about 200 patients in hospital-based MRI or autopsy series.^1^, ^2^, ^3^, ^4^, ^5^ Population-based annual CCM detection rates were 0·17 (95% CI 0·00–0·34) per 100 000 people in the USA from 1965 to 1992 compared with 0·56 (0·41–0·75) per 100 000 in Scotland from 1999 to 2000;^6^, ^7^ this difference in detection rates might be partly explained by the increasing availability and use of brain MRI. CCMs can be sporadic or inherited as an autosomal dominant trait,^8^ in which multiple CCMs occur, and appear de novo.^9^

**Figure 1:**
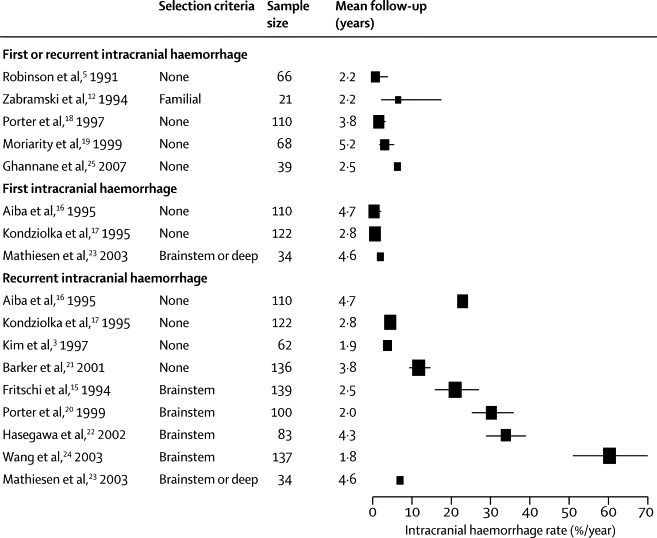
Risk of symptomatic intracranial haemorrhage during follow-up in studies of the untreated clinical course of over 20 participants with cerebral cavernous malformations Areas of point estimates are proportional to the sample size of each study. Error bars represent 95% CIs (if available or calculable).

CCMs are blood vessels devoid of muscular and elastic tissue that are lined with endothelial cells that do not have intervening tight junctions. CCMs are prone to haemorrhage,^10^ which results in distinctive diagnostic appearances on pathological examination and MRI.^10^, ^11^, ^12^ Of CCMs diagnosed in adults on the basis of neurological symptoms, one quarter are identified owing to intracranial haemorrhage and another quarter are identified after a focal neurological deficit without radiographic evidence of recent haemorrhage;^13^ the remainder of patients present with epileptic seizures.^14^ Hospital-based case series have described the untreated clinical course of CCMs during mostly retrospective observation, with means of 1·9–5·2 years follow-up. In these studies, inception points (ie, the start of follow-up), diagnostic criteria, outcome definitions, and methods of assessment and analysis varied (figure 1 and webappendix pp 1–3).^3^, ^5^, ^12^, ^15^, ^16^, ^17^, ^18^, ^19^, ^20^, ^21^, ^22^, ^23^, ^24^, ^25^ In these studies, the annual risk of first intracranial haemorrhage (range 0·4–0·6%)^16^, ^17^ and the annual risk of recurrent intracranial haemorrhage (3·8–22·9%)^3^, ^16^, ^17^, ^21^ varied (figure 1). Patients with brainstem CCM seem to have a higher risk of recurrent intracranial haemorrhage when indirectly compared with cohorts of patients with CCM in other brain regions (21·0–60·2%; figure 1),^15^, ^20^, ^22^, ^24^ although internal comparisons within individual cohorts have not confirmed this finding.^17^, ^21^ Findings have not been consistent regarding whether female sex is associated with incident haemorrhage^5^, ^16^ or not,^19^ or with recurrent intracranial haemorrhage^16^, ^19^ or not.^17^, ^18^, ^21^ Intracranial haemorrhage from a CCM tends to be intracerebral and of low volume,^26^ although case fatality has ranged from 0% overall^18^ to 17% for recurrent haemorrhage from brainstem CCMs.^15^ Little is known about the effect of intracranial haemorrhage or non-haemorrhagic focal neurological deficits on survivors' functional outcome.^18^, ^27^, ^28^

Therefore, we investigated the risks, predictors, and functional effect of intracranial haemorrhage and non-haemorrhagic focal neurological deficits in a prospective, population-based cohort study. We planned to report outcomes at 5 years to encourage standardisation of outcome reporting for CCMs,^13^ and we aimed to stratify survival analyses by mode of initial CCM presentation. We hypothesised that female sex and brainstem CCM location would also predict poor outcome.

## Methods

### Patients

The Scottish Audit of Intracranial Vascular Malformations (SAIVMs) is an ongoing National Health Service clinical audit of adults who were aged 16 years or older and were resident in Scotland when first diagnosed with any type of intracranial vascular malformation during 1999–2003 and 2006–2010. The Scottish Intracranial Vascular Malformations Study (SIVMS) is a prospective, population-based cohort study in which we used anonymous data extracts from SAIVMs. We have published the audit protocol and registered the research protocol with the Directory of Clinical Databases (DoCDat). Patients were identified through multiple overlapping sources of case ascertainment, which included a Scotland-wide collaborative network of neurologists, neurosurgeons, stroke physicians, radiologists, and pathologists and central registers of hospital discharge records and death certificates.^7^ In this analysis, we included every adult in SIVMS who had a first-in-a-lifetime definite diagnosis of CCM in the years 1999–2003, made on the basis of pathological examination or brain MRI.^11^, ^12^

The Multicentre Research Ethics Committee for Scotland (MREC/98/0/48) and the Fife and Forth Valley Research Ethics Committee (08/S0501/76) approved the undertaking of observational studies (to which an opt-out consent policy applied) and postal questionnaire studies (which required opt-in consent).

### Procedures

The inception point was an adult's initial presentation, which was the date of symptom onset or medical consultation (if asymptomatic) that led to an investigation that diagnosed a CCM. We used annual surveillance of family doctor and hospital medical records, as well as annual postal questionnaires to family doctors and consenting participants with a CCM on each anniversary of CCM diagnosis, to establish demographics and medical histories, to identify outcome events, and to assess adults' dependence (by the Oxford Handicap Scale [OHS]) prospectively during follow-up.^29^

Two neuroradiologists (JJB and RJS) used the diagnostic brain images that had been obtained in clinical practice to verify CCM diagnosis with reference to accepted criteria^11^, ^12^ and collected data on CCM anatomical location, CCM size, coexistent intracranial vascular malformations, and radiological evidence for recent intracranial haemorrhage.^13^ We also reviewed brain imaging and reports of pathological examinations to classify the mode of initial CCM presentation. Two investigators (CPW and RA-SS) assessed outcome events using the clinical, radiological, and pathological information available, masked to sex and CCM location. To attribute the mode and cause of death, we reviewed death certificates, autopsy reports if post-mortem examination had been done, and clinical records and pertinent brain images if death had occurred during a hospital stay.

We used published criteria to distinguish intracranial haemorrhage, non-haemorrhagic focal neurological deficits (if timely brain imaging of the appropriate modality had not identified fresh haemorrhage), and focal neurological deficits not otherwise specified (if timely brain imaging of the appropriate modality had not been done).^13^ We regarded initial presentations as incidental if the adult had been asymptomatic or if their symptoms (eg, headache) could not be related to the underlying CCM. We attributed initial presentations to epileptic seizures if the seizures were not symptomatic of a concomitant intracranial haemorrhage. When assessing clinical events at initial presentation and during follow-up, we also classified whether they were definitely, possibly, or definitely not attributable to the CCM. We classified events as possibly attributable to a CCM when the clinical features of an event were anatomically consistent with CCM location, but another cause (eg, ischaemic stroke) was possible and neuroradiological investigation had not identified either CCM haemorrhage or an alternative cause.

The primary outcome was a composite of intracranial haemorrhage or focal neurological deficit because their severities seem to be similar^26^ and many focal neurological deficits might be haemorrhages undetected by imaging.^13^ We quantified intracranial haemorrhage alone to facilitate comparison with other studies, but chose a composite primary outcome to show all the neurological deficits experienced by patients. The primary outcome combined events definitely attributable to a CCM with those possibly attributable because of the absence of a better alternative explanation.^13^ We have described the occurrence of seizures after a diagnosis of CCM in this cohort elsewhere.^14^

### Statistical analysis

We categorised CCM location as brainstem (in the midbrain, pons, or medulla), cerebellar, deep (in the thalamus or basal ganglia), or lobar (in the cortex or subcortical areas of the cerebral hemispheres). We dichotomised location into brainstem versus other locations for univariate analyses; if an adult had many CCMs, we allocated a primary location according to the location of the symptomatic CCM, but in asymptomatic adults the brainstem CCM location took precedence because it was postulated to be a predictor of intracranial haemorrhage or focal neurological deficit. We used parametric statistics for between-group comparisons when the data obeyed a normal distribution and non-parametric statistics when they did not. We used exact tests when cell counts were fewer than five.

We quantified completeness of the follow-up data we had accrued as a proportion of all the follow-up that could have been obtained before death or the end of the 5-year timeframe for these analyses.^30^ We used life tables and Kaplan-Meier estimates together with log-rank tests to analyse follow-up data accrued by February, 2011. Survival analyses of time to first event for adults who were event-free at presentation started at the date of initial presentation and stopped at the date of the first outcome event or the date of censoring, whichever occurred first. Survival analyses of time to earliest recurrent event started at the date of the first event (whether it occurred at initial presentation or during follow-up) and stopped at the date of the earliest recurrent outcome or the date of censoring, whichever occurred first. We censored follow-up at the earliest occurrence of any of the following: death unrelated to CCM, first CCM treatment (with surgical excision or stereotactic radiosurgery, at the discretion of the treating physician), last available follow-up, or 5 years after initial presentation.

We stratified survival analyses by mode of initial CCM presentation. We investigated the effect of two potential predictors: men versus women and brainstem versus other CCM primary locations. We prespecified these factors on the basis of their clinical relevance and their hypothesised effect on outcome, as well as on the accuracy, reliability, and completeness of their ascertainment. We did univariate comparisons with the log-rank test, quantified survival functions at 5 years, and did Cox regression if proportional hazards assumptions were satisfied.^31^ We used sensitivity analyses to assess whether restriction of analyses to events definitely attributable to CCM affected our primary outcome analyses.

We did not prespecify our desired sample size, but instead we sought to identify every new definite CCM diagnosis over 5 years in one country (the mid-2010 population estimate of adults aged 16 years or older was 4·31 million)^32^ and accrue follow-up until we had sufficient outcome events to analyse our two potential predictors in multivariable analyses (at least 20 events to fit a multivariable model with two covariates). We used two-tailed statistical tests (α=0·05). Analyses were done with SPSS (version 16.0), Stata (version 11.2), StatsDirect (version 2.7.8), and Confidence Interval Analysis software (version 2.2.0).

### Role of the funding source

The study sponsors had no role in study design, in the collection, analysis, and interpretation of data, in the writing of the report, or in the decision to submit the paper for publication. The corresponding author had full access to all study data and had final responsibility for the decision to submit for publication.

## Results

From 1999 to 2003, 139 adult residents in Scotland were newly diagnosed with at least one definite CCM (133 on brain MRI, five at autopsy, and one after surgical excision). Of these 139 adults, 24 had multiple CCMs, 20 had associated developmental venous anomalies, two had an unrelated intracranial aneurysm, and one had an unrelated brain arteriovenous malformation.

The median age of the 139 adults at the initial presentation that led to CCM diagnosis was 41 years (IQR 32–53 years) and 80 (58%) were women. The symptoms leading to CCM diagnosis were incidental (n=66, 47%), epileptic seizure (n=35, 25%), intracranial haemorrhage (n=17, 12%), and focal neurological deficits (n=21, 15%). The primary CCM locations were lobar (n=93, 67%), brainstem (n=19, 14%), cerebellar (n=18, 13%), and deep (n=9, 6%), and there was no association between female sex and CCM location (χ^2^ test, p=0·7).

After omitting the five adults whose CCM (two brainstem and three lobar) were first diagnosed incidentally at autopsy and who did not contribute to our outcome analyses, we compared 134 adults according to their type of initial presentation (table). Adults were older at the time of incidental CCM detection than at symptomatic initial presentations (Kruskal Wallis test, p=0·007). Adults initially presenting with intracranial haemorrhage or focal neurological deficit were more likely to harbour brainstem CCMs (32% *vs* 5% with other modes of presentation, Fisher's exact test p<0·0001) and seemed to be more likely to be women, although this was not statistically significant (71% *vs* 54%, χ^2^ test, p=0·07).

**Table:** Baseline characteristics of adults who were alive at the time of their diagnosis of cerebral cavernous malformations

		**Incidental presentation (n=61)**	**Presentation with epileptic seizure or seizures (n=35)**	**Presentation with ICH or FND (n=38)**
Age (years)		45 (34–54)	34 (26–46)	38·5 (32·5–56)
Women		38 (62%)	14 (40%)	27 (71%)
Primary CCM location				
	Lobar	42 (69%)	35 (100%)	13 (34%)
	Deep	4 (7%)	0 (0%)	5 (13%)
	Cerebellum	10 (16%)	0 (0%)	8 (21%)
	Brainstem	5* (8%)	0 (0%)	12 (32%)
Multiple CCMs		6 (10%)	11 (31%)	6 (16%)
Associated developmental venous anomaly		11 (18%)	1 (3%)	8 (21%)

Data are median (IQR) or number (%). CCM=cerebral cavernous malformation. ICH=intracranial haemorrhage. FND=focal neurological deficit.

* Four adults with incidental multiple CCM were allocated a primary brainstem location on the basis of the existence of at least one brainstem CCM.

**Figure 2:**
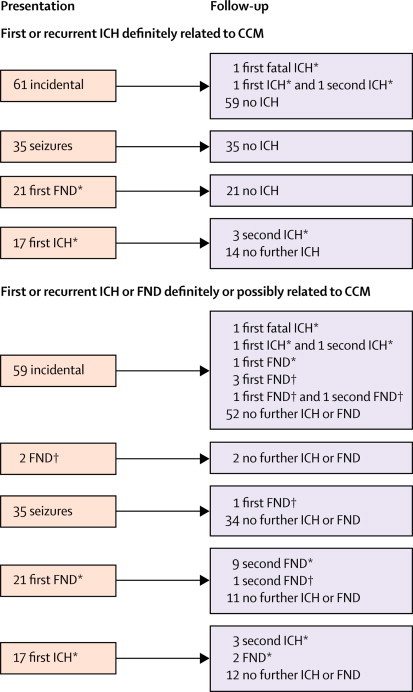
Flowcharts showing the outcomes of adults included in the analyses ICH=intracranial haemorrhage. FND=focal neurological deficit. *Event definitely related to cerebral cavernous malformation. †Event possibly related to cerebral cavernous malformation.

We followed up the 134 adults with CCM who were alive at initial presentation for 1177 person-years (of 1216 potential person-years, for an overall completeness of 97%).^30^ We limited our analyses to the first 5 years of follow-up (although years 6 and 7 of follow-up were available for this cohort, no outcome events occurred during that time). In these analyses, the median duration of follow-up per adult was 5 years; of the 17 adults followed up for less than 5 years, 15 died within the 5-year period. Follow-up ended for these 134 adults because of censoring at last follow-up (n=97), CCM treatment (n=23, surgical excision in all cases), or death unrelated to CCM (n=14). Figure 2 describes the grouping of adults in our analyses.

**Figure 3:**
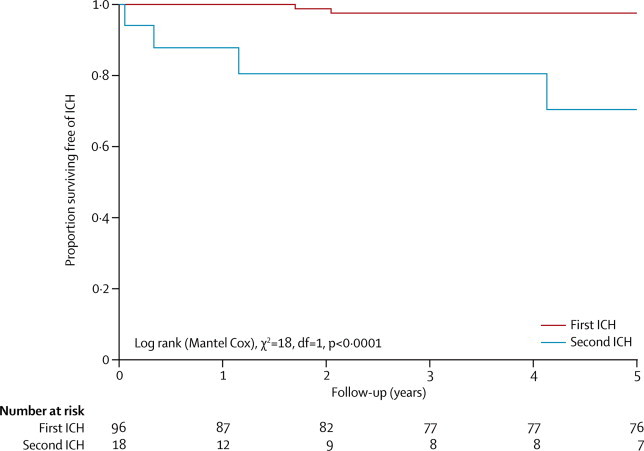
Kaplan-Meier estimates of progression to first or second intracranial haemorrhage definitely attributable to cerebral cavernous malformation ICH=intracranial haemorrhage.

Of 96 adults who initially presented incidentally or with seizures, one man and one woman had a first intracranial haemorrhage (5-year risk 2·4%, 95% CI 0·0–5·7; figure 3). Taking together the one survivor of these first intracranial haemorrhages during follow-up and the 17 adults with first intracranial haemorrhage at initial presentation, four adults (all women) had a second intracranial haemorrhage (5-year risk 29·5%, 4·1–55·0; figure 3), which was greater than the risk of a first intracranial haemorrhage (log-rank p<0·0001). Of all 23 instances of a first or second haemorrhage, one was followed by death within 30 days from a first intracranial haemorrhage caused by a lobar CCM (case fatality 4·3%, 95% CI 0·8–21·0).

**Figure 4:**
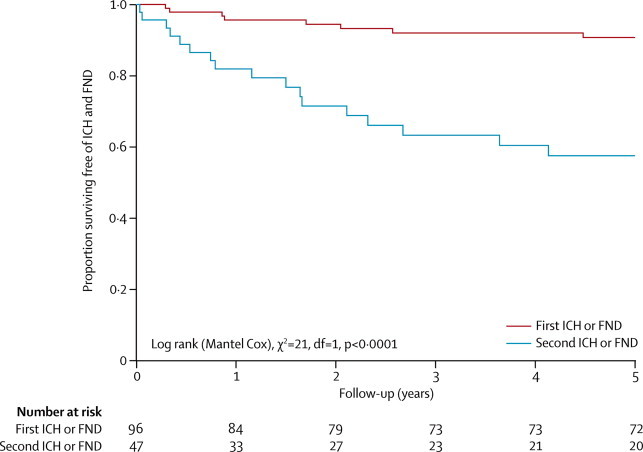
Kaplan-Meier estimates of progression to first or second intracranial haemorrhage or focal neurological deficit definitely or possibly attributable to cerebral cavernous malformation ICH=intracranial haemorrhage. FND=focal neurological deficit.

For the primary outcome of intracranial haemorrhage and focal neurological deficits (not including epileptic seizure) that were definitely or possibly related to CCM, eight of the 96 adults who initially presented incidentally or with seizures had a first event (5-year risk 9·3%, 95% CI 3·1–15·4; figure 4). However, of the 47 adults who had, at presentation or during follow-up, a non-fatal first intracranial haemorrhage or focal neurological deficit that was definitely or possibly related to CCM, 17 had a second event (5-year risk 42·4%, 95% CI 26·8–58·0; figure 4), which was greater than the risk of a first event (log-rank p<0·0001). These findings were unchanged in a sensitivity analysis that excluded the events that were possibly attributable to CCM (webappendix p 4). Three of the 17 adults who had a second event had further recurrences during the follow-up period. The annual risk of a second intracranial haemorrhage or focal neurological deficit that was definitely or possibly related to CCM declined during follow-up: 19·8% (95% CI 6·1–33·4) in year 1, 13·3% (0·0–26·4) in year 2, 12·0% (0·0–25·6) in year 3, 4·5% (0·0–13·5) in year 4, and 5·0% (0·0–14·8) in year 5. We could not detect any statistically significant difference in functional outcome: after 5 years, there was no difference in the proportion of adults scoring 0–2 on the OHS after first (89%) or second (100%) intracranial haemorrhages or focal neurological deficits that were definitely or possibly related to CCM (webappendix p 5).

**Figure 5:**
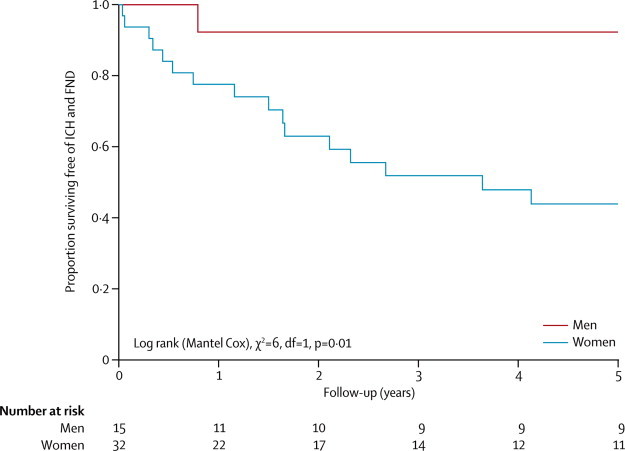
Sex-stratified Kaplan-Meier estimates of progression to second intracranial haemorrhage or focal neurological deficit definitely or possibly attributable to cerebral cavernous malformation ICH=intracranial haemorrhage. FND=focal neurological deficit.

We explored our two prespecified potential predictors of a second event for the 47 adults who had a first intracranial haemorrhage or focal neurological deficit that was definitely or possibly attributable to CCM. The risk of a second event was significantly higher for women than men (figure 5; p=0·01). In a post-hoc analysis of the influence of sex on recurrent intracranial haemorrhage alone, we found that all four recurrent haemorrhages occurred in women but that the difference in risk between men and women was not statistically significant (p=0·29). We did not identify a greater risk for brainstem CCM compared with CCM in other locations (p=0·17; webappendix p 6). We were unable to do Cox regression involving both sex and CCM location because proportional hazards assumptions were not fulfilled (webappendix p 7).^31^ We assessed the potential for, but could not confirm, confounding between sex and CCM location among these 47 adults: of 12 women with brainstem CCM, seven (58%) had outcome events; of two men with brainstem CCM, one (50%) had an outcome event; of 20 women with non-brainstem CCM, nine (45%) had outcome events; and of 13 men with non-brainstem CCM, none (0%) had an outcome event. Multiple CCMs (*vs* solitary CCM; p=0·7) and the presence of an associated developmental venous anomaly (*vs* the absence of such an anomaly; p=0·3) did not predict a second event during follow-up (data not shown).

## Discussion

In this prospective, population-based study of adults with CCM, the 5-year risk of a first intracranial haemorrhage was lower than the risk of recurrence. The difference between the risks of first and recurrent events was also evident for the primary composite endpoint, for which the annual risk of intracranial haemorrhage or focal neurological deficit definitely or possibly related to CCM was higher for women than men and declined significantly over 5 years. We confirmed our prespecified hypothesis that sex influences the risk of recurrence.

In this study, we minimised several potential sources of bias: selection bias, by using a population-based design restricted to newly diagnosed cases; detection and misclassification biases, by using strict diagnostic criteria and outcome definitions;^11^, ^13^ information bias, by using prospective follow-up, which attained 97% completeness over 5 years; and bias in outcome assessment, by masking assessors to potential prognostic features.^33^, ^34^, ^35^ We followed up each adult for a median of 5 years and quantified outcomes at 5 years to facilitate comparison with future studies;^13^ we could have quantified outcomes over 7 years, but no outcome events occurred between 5 and 7 years, which further underscores our finding of a diminishing annual rate of recurrence over time.

Despite identifying all incident CCM diagnoses in an adult population over 5 years and assessing the cohort for 5 years, the precision of our estimates could be improved, and we have addressed this by recently identifying a second CCM cohort, results from which will be reported in the future. We used clinical information and the uncertainties of symptom attribution to CCM inherent in everyday clinical practice to enhance the generalisability of our findings,^33^, ^34^, ^35^ but we might have missed some events by not relying on scheduled study visits. Anecdotally, CCM haemorrhage can present with epileptic seizure alone, so we might have underestimated CCM haemorrhage rates, but brain imaging is not used to investigate every seizure in clinical practice. Survival analyses include an assumption that censoring is not informative, but treatment might be related to a patient's future prognosis. In some of our comparisons with log-rank tests, a few adults contributed different periods of their follow-up time to the two groups being compared. The tendency of clinicians to investigate young, normotensive patients with intracerebral haemorrhage could have led to bias in CCM detection in favour of these groups.^36^ The classification of presenting and outcome events as either intracranial haemorrhage or focal neurological deficit depends on the availability and use of timely imaging of the appropriate modality, which varies between health services. To facilitate comparisons with other cohorts, and because both of these clinical events are of comparable clinical effect and probably share the same pathophysiology, we amalgamated them in a composite outcome, but work remains to be done on the inter-rater and intra-rater reliability of the classification of these outcomes.^13^

The difference in risk of recurrence by sex might be explained by reporting bias but, as has been found in other contexts,^37^ we think a true difference is a more likely explanation, which might also have caused the possible preponderance of women initially presenting with intracranial haemorrhage or focal neurological deficit. Various explanations for this finding involve biologically plausible mechanisms related to the hormonal responsiveness of CCM, due to pregnancy, contraception, or hormone replacement therapy.^5^, ^16^, ^20^, ^38^

Most published studies of CCM prognosis have been small, single-centre case series that were potentially subject to selection bias, which might explain some of the differences between these studies (panel). In some studies, lifetime event rates have been calculated retrospectively assuming CCM presence since birth, but we did not include these studies in our systematic review or use this technique because CCMs are known to occur de novo and might not be congenital.^9^ Most published studies have combined first and recurrent events and calculated annualised risks without actuarial analysis during follow-up,^5^, ^12^, ^18^, ^19^, ^25^ which masked the phenomenon of diminishing event rate and temporal clustering of CCM haemorrhages.^21^, ^24^

Panel **Research in context**

**Systematic review**

We used electronic strategies (webappendix p 1) to search for journal articles, published before Dec 1, 2011, and indexed in OVID Medline and Embase, that described original studies of more than 20 adults with cerebral cavernous malformations (CCMs) and enumerated symptomatic intracranial haemorrhages during a quantified period of follow-up. We systematically compared these studies with our characteristics of an ideal study of CCM prognosis (webappendix pp 2–3).^33^, ^34^, ^35^ We extracted the published annualised symptomatic intracranial haemorrhage rate from each study (or calculated it on the basis of the number of symptomatic intracranial haemorrhages occurring during its total person-years of follow-up after CCM diagnosis). We also extracted the corresponding 95% CI if published (or calculated the 95% CI around this incidence rate with Confidence Interval Analysis software, if the necessary data had been published), and stratified our presentation of these rates in the 14 included studies by mode of initial CCM clinical presentation (figure 1).^3^, ^5^, ^12^, ^15^, ^16^, ^17^, ^18^, ^19^, ^20^, ^21^, ^22^, ^23^, ^24^, ^25^

**Interpretation**

Published studies have quantified variable outcomes for adults with CCM according to sex, mode of initial presentation, and CCM location (figure 1). To our knowledge, this is the first cohort study of CCM that is prospective, is population-based, and adheres to standards recommended for prognosis research.^11^, ^12^, ^13^, ^33^, ^34^, ^35^ We show that the risk of recurrent intracranial haemorrhage is an order of magnitude greater than the risk of a first haemorrhage, and that the risk of a recurrent intracranial haemorrhage or focal neurological deficit is greater for women than men and declines over time.

Our findings are important for clinical practice, in which patients and their clinicians have to make decisions about CCM treatment on the basis of the untreated clinical course of CCM and without the benefit of randomised controlled trials. We have shown that the risk of a first-ever intracranial haemorrhage is low; functional impairment from haemorrhage is mild at initial presentation,^26^ and from our findings it seems that further recovery occurs during follow-up and 30-day case fatality is low. Although the risk of recurrence is higher than the risk of a first event, especially for women, the risk of recurrence seems to decline over time. This decline provides some reassurance for patients as time passes without CCM treatment after a haemorrhage, and suggests that decision making about CCM treatment in clinical practice (and in future randomised controlled trials) might not need to be compared with the untreated clinical course in the long term when event rates may be very low.

We, and others, should continue observation of these patients over their lifetimes to confirm the apparent decline in risk of recurrent intracranial haemorrhage or focal neurological deficit over time.^21^, ^24^ Greater precision is also needed: the size of our cohort should double with the addition of adults diagnosed from 2006 to 2010, and we are planning an individual patient data meta-analysis with other cohorts. These further studies might improve the precision of our estimates and allow confirmation of the predictors that we have identified, assessment of other potential predictors, and investigation of why women are at a higher risk of recurrence than men.^34^ The 5-year event rates and the declining risk of recurrence that we have observed will help in the design of randomised controlled trials, which hitherto have not been conducted for CCM.

## Web Extra Material

Supplementary webappendix
