# Supplementary material for: Untreated clinical course of cerebral cavernous malformations: a prospective, population-based cohort study
Source: Lancet Neurol. 2012 Mar;11(3):150–6. doi: 10.1016/S1474-4422(12)70004-2 (PMC3282211; doi:10.1016/S1474-4422(12)70004-2)
Supplement: Supplementary webappendix [file mmc1.pdf]

THE LANCET Neurology

Supplementary webappendix

This webappendix formed part of the original submission and has been peer reviewed. We post it as supplied by the authors.

Supplement to: Al-Shahi Salman R, Hall JM, Horne MA, et al, for the Scottish Audit of Intracranial Vascular Malformations (SAIVMs) collaborators. Untreated clinical course of cerebral cavernous malformations: a prospective, population-based cohort study. *Lancet Neurol* 2012; published online Jan 31. DOI:10.1016/S1474-4422(12)70004-2.

WEBAPPENDIX 1 – Literature search strategies

MEDLINE

1. Hemangioma, Cavernous, Central Nervous System/
2. Hemangioma, Cavernous/
3. (cavernous adj5 (angioma$ or hemangioma$ or malformation$)).tw.
4. cavernoma$.tw.
5. 2 or 3 or 4
6. exp brain/ or central nervous system/ or exp cerebral arteries/
7. exp brain neoplasms/
8. (brain$ or cerebral or intracerebral or central nervous system or intracranial or cerebellar or intraventricular or supratentorial).tw.
9. 6 or 7 or 8
10. 5 and 9
11. 1 or 10

EMBASE

1. Brain Hemangioma/
2. brain ventricle cavernoma/
3. cavernous hemangioma/
4. (cavernous adj5 (angioma$ or hemangioma$ or malformation$)).tw.
5. cavernoma$.tw.
6. 3 or 4 or 5
7. central nervous system/ or exp brain/ or exp brain ventricle/ or exp brain artery/
8. exp brain tumor/
9. (brain$ or cerebral or intracerebral or central nervous system or intracranial or cerebellar or intraventricular or supratentorial).tw.
10. 7 or 8 or 9
11. 6 and 10
12. 1 or 2 or 11

WEBAPPENDIX 2 – Selection of studies of the untreated clinical course of adults with cerebral cavernous malformation(s).[4-6]

IDENTIFICATION

Electronic database searches, after de-duplication
(n= 1,445)

SCREENING

Titles and abstracts screened
(n= 1,445)

→ **Studies excluded**
(n= 1,417)

ELIGIBILITY

Full text articles assessed for eligibility
(n= 28)

Assessment of ideal characteristics

- Complete sample (ideally population-based, with selection criteria described)
- Radiological or pathological diagnostic certainty
- Inception cohort, defined at a clear time point in the disease
- Prospective data collection
- Sample size >20 *
- Period at risk from first CCM diagnosis or symptoms leading to it, enabling calculation of event rates (not retrospective 'lifetime risk') *
- Outcome events
  - Objective, symptomatic clinical outcome events[1]
    - *Case fatality*
    - *Intracranial haemorrhage **
    - *Standard measure of disability (e.g. modified Rankin Scale)*
  - Assessed independently, blind to prognostic factors of interest
- Follow-up
  - Censored at the time of treatment, if treated
  - Available for ≥90% of the cohort
  - Duration >1 year
  - Outcome events quantified per patient during a specified follow-up interval *

** Requirements for inclusion in this systematic review*

→ **Full text articles excluded** (n=14)

- CCM diagnosis was not certain[2,3]
- Retrospective lifetime period at risk[10-14]
- Outcome events were not objective and symptomatic[18-20]
- Duration of follow-up was not adequately quantified[24-27]

INCLUDED

Studies of untreated clinical course of CCM
(n=14) [7-9,15-17,21-23,28-32]

WEBAPPENDIX 3 – Characteristics of studies of the untreated clinical course of >20 participants with cerebral cavernous malformation(s).

Study	Diagnostic certainty	Inception cohort	Prospective	Objective clinical outcomes	Follow-up period censored at treatment	Follow-up ≥ 90%	>1 year follow-up for all participants
Robinson *et al* 1991 [7]	■	□	□	■	?	□	?
Fritschi *et al* 1994 [8]	?	?	□	■	■	■	□
Zabramski *et al* 1994 [9]	■	□	■	■	?	■	■
Aiba *et al* 1995 [15]	■	■	□	■	■	■	□
Kondziolka *et al* 1995 [16]	■	□	■+□	?	■	□	□
Kim *et al* 1997 [17]	■	?	□	?	■	?	■
Porter *et al* 1997 [21]	■	■	■+□	■	■	■	□
Moriarity *et al* 1999 [22]	■	■	■	■	?	?	?
Porter *et al* 1999 [23]	■	□	□	■	?	■	□
Barker *et al* 2001 [28]	■	■	□	■	■	?	?
Hasegawa *et al* 2002 [29]	■	■	■+□	■	■	?	□
Mathiesen *et al* 2003 [30]	?	?	■+□	?	■	?	?
Wang *et al* 2003 [31]	■	■	?	■	■	■	□
Ghannane *et al* 2007 [32]	■	?	□	■	■	■	□

■ = characteristic fulfilled; □ = characteristic not fulfilled; ? = unclear / not specified

WEBAPPENDIX 4 – Flowcharts of the adults with cerebral cavernous malformation(s) contributing to analyses in this cohort study.
ICH = intracranial haemorrhage. FND = focal neurological deficit. * = Event definitely related to CCM. † = event possibly related to CCM.

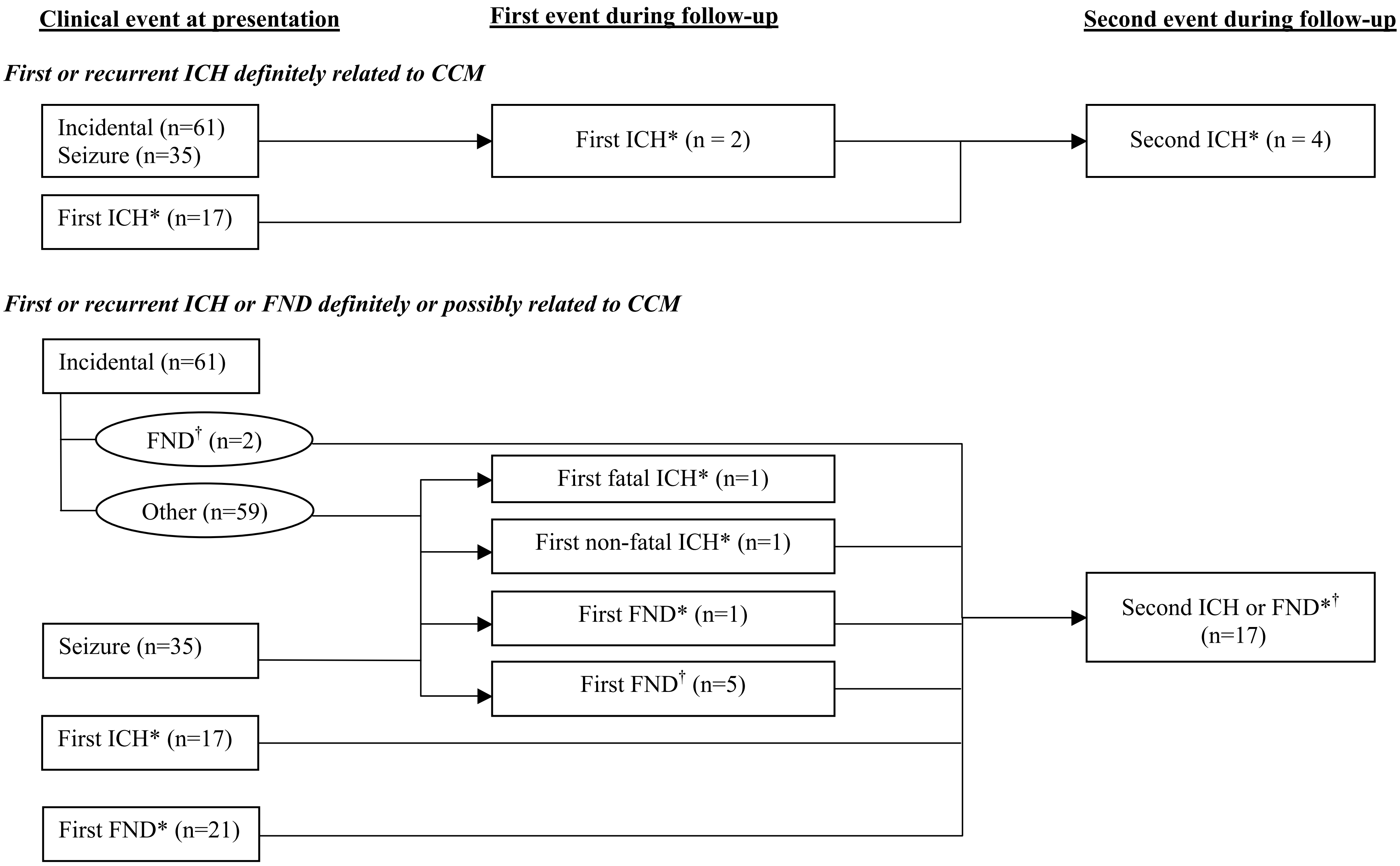

WEBAPPENDIX 5 – Kaplan Meier estimates of progression to first or second intracranial haemorrhage or focal neurological deficit definitely attributable to cerebral cavernous malformations during five years of prospective, untreated follow-up.

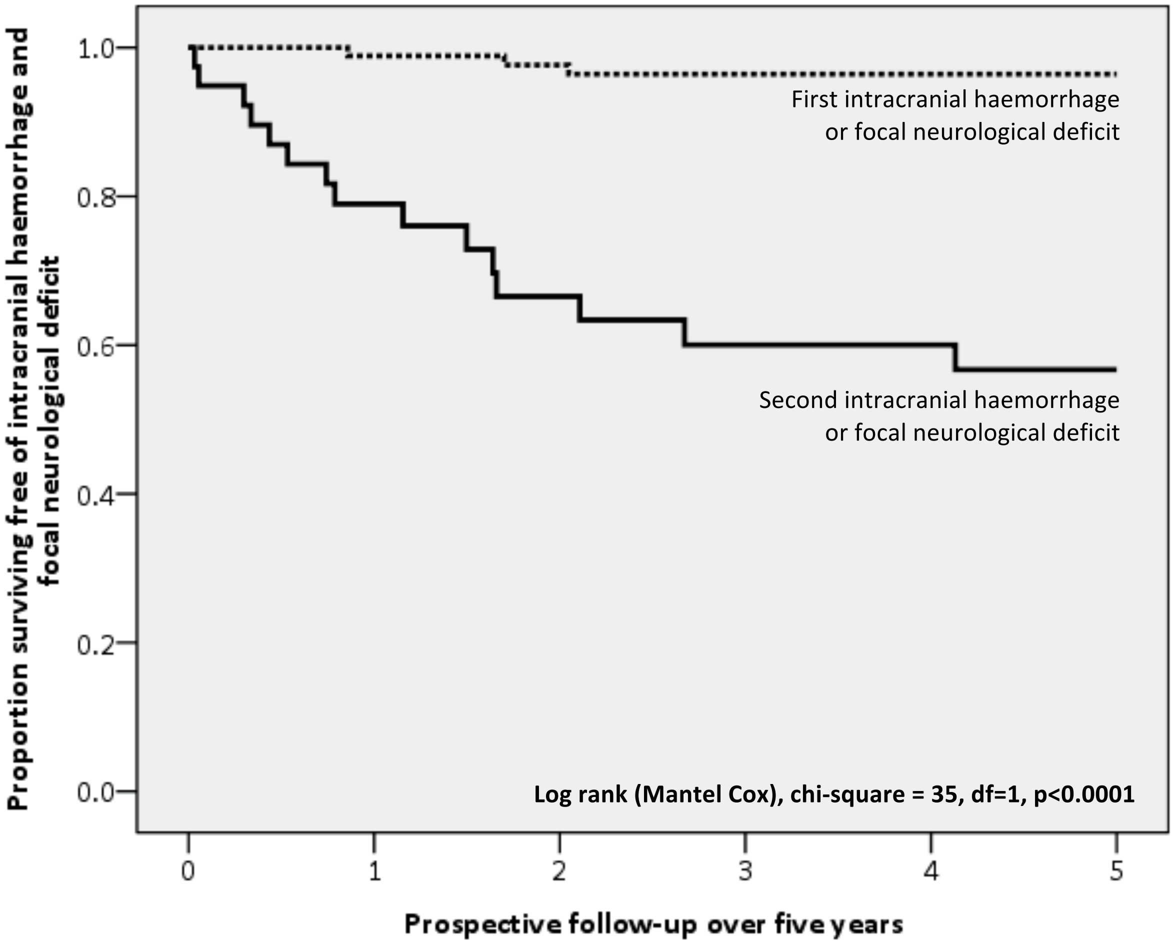

**Adults remaining**						
·········	96	86	81	76	76	75
———	40	27	21	18	18	17

WEBAPPENDIX 6 – Dependence measured on the Oxford Handicap Scale for survivors of first and second intracranial haemorrhage or focal neurological deficit. Data are provided from annual postal questionnaires, until the time of treatment (23 adults underwent surgical excision).

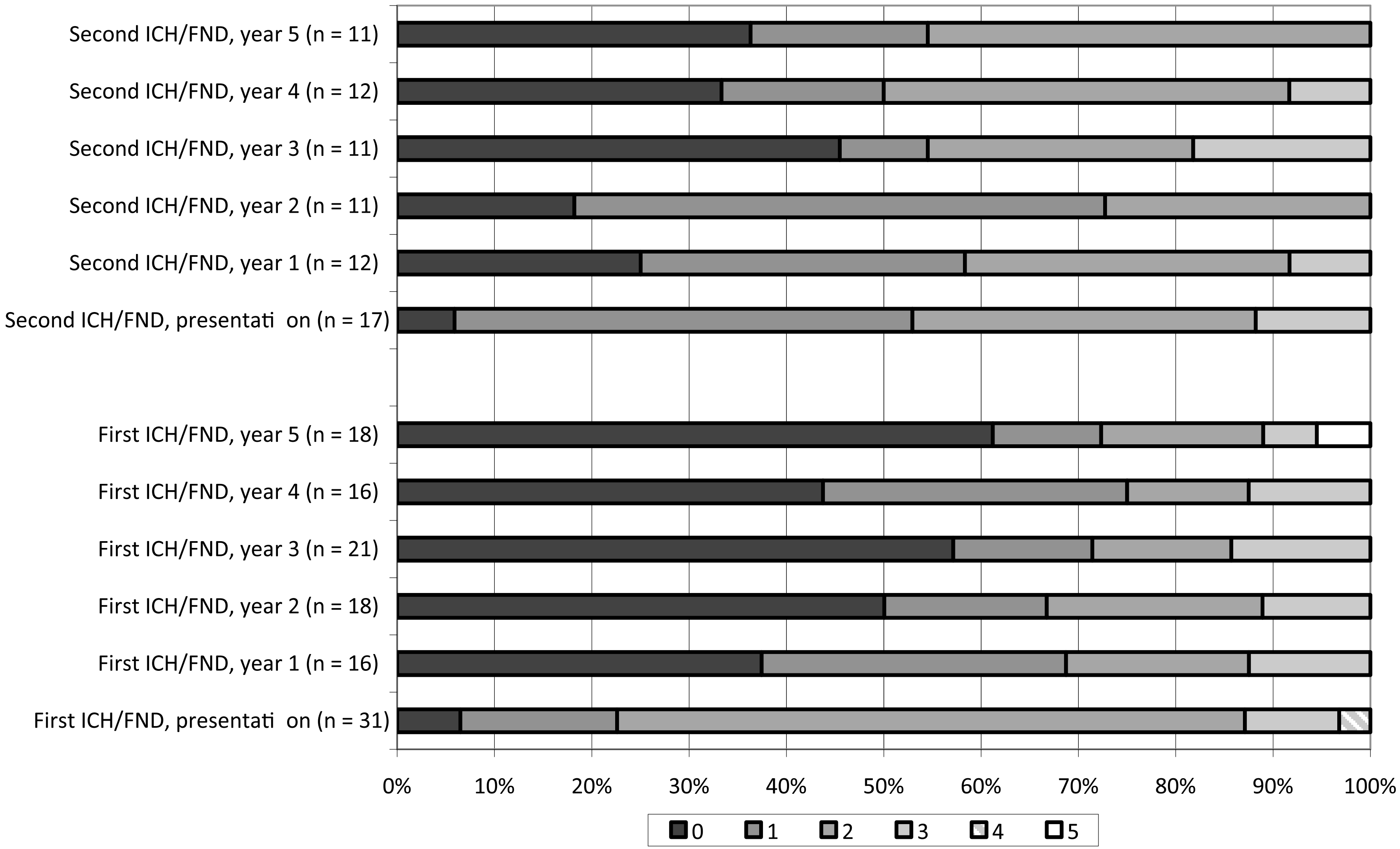

WEBAPPENDIX 7 – Kaplan Meier estimates of progression to second intracranial haemorrhage or focal neurological deficit, stratified by brainstem versus non-brainstem location, for adults with cerebral cavernous malformation(s) during five years of prospective, untreated follow-up.

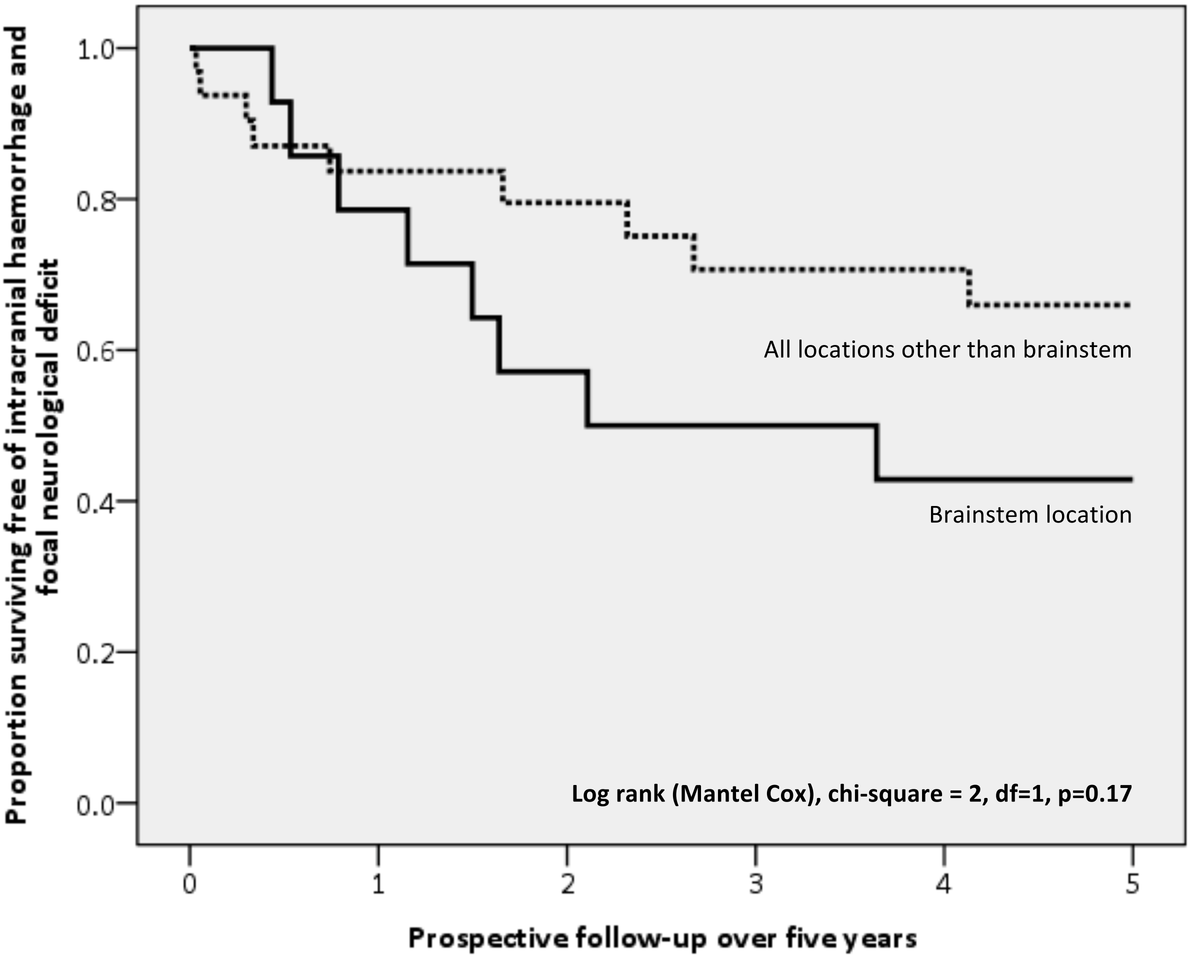

Adults remaining						
··········	33	22	19	16	15	14
———	14	11	8	7	6	6

WEBAPPENDIX 8 – Assessment of whether cerebral cavernous malformation location and sex fulfilled proportional hazards assumptions.

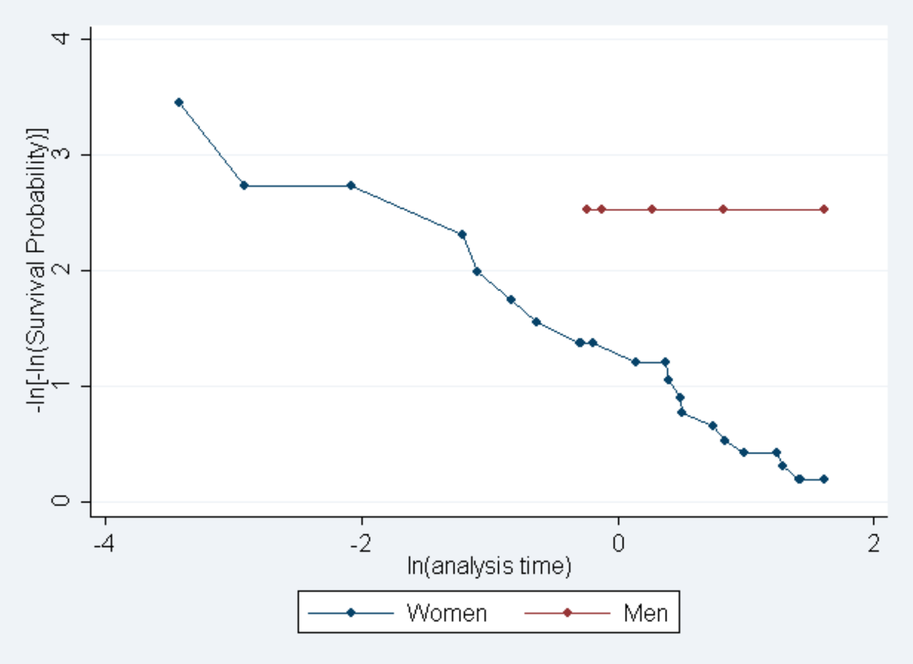

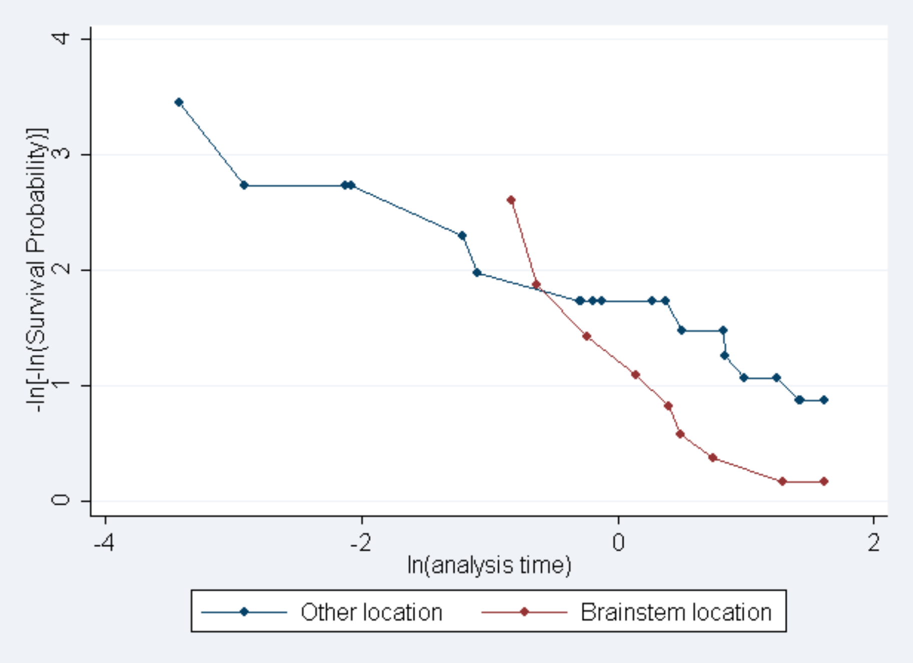

REFERENCES

(1) Al-Shahi Salman R, Berg MJ, Morrison L, Awad IA, Angioma Alliance Scientific Advisory Board. Hemorrhage from cavernous malformations of the brain: definition and reporting standards. Stroke 2008; 39(12):3222-3230.

(2) Abe M, Kjellberg RN, Adams RD. Clinical presentations of vascular malformations of the brainstem:comparison of angiographically positive and negative types. J Neurol Neurosurg Psychiatry 1989; 52:167-175.

(3) Lobato RD, Perez C, Rivas JJ, Cordobes F. Clinical, radiological, and pathological spectrum of angiographically occult intracranial vascular malformations. Analysis of 21 cases and review of the literature. J Neurosurg 1988; 68(4):518-531.

(4) Raymond J, Naggara O, Guilbert F, Altman DG. Assessing prognosis from nonrandomized studies: an example from brain arteriovenous malformations. AJNR 2011; 32(5):809-812.

(5) Hemingway H, Riley RD, Altman DG. Ten steps towards improving prognosis research. Br Med J 2009; 339:b4184.

(6) Grimes DA, Schulz KF. Cohort studies: marching towards outcomes. Lancet 2002; 359(9303):341-345.

(7) Robinson JR, Awad IA, Little JR. Natural history of the cavernous angioma. J Neurosurg 1991; 75(5):709-714.

(8) Fritschi JA, Reulen HJ, Spetzler RF, Zabramski JM. Cavernous malformations of the brain stem. A review of 139 cases. Acta Neurochir (Wien) 1994; 130(1-4):35-46.

(9) Zabramski JM, Wascher TM, Spetzler RF, Johnson B, Golfinos J, Drayer BP et al. The natural history of familial cavernous malformations: results of an ongoing study. J Neurosurg 1994; 80(3):422-432.

(10) Bruneau M, Bijlenga P, Reverdin A, Rilliet B, Regli L, Villemure JG et al. Early surgery for brainstem cavernomas. Acta Neurochir (Wien) 2006; 148(4):405-414.

(11) Ferroli P, Sinisi M, Franzini A, Giombini S, Solero CL, Broggi G. Brainstem cavernomas: long-term results of microsurgical resection in 52 patients. Neurosurgery 2005; 56(6):1203-1212.

(12) Del Curling O. Jr, Kelly DL, Jr., Elster AD, Craven TE. An analysis of the natural history of cavernous angiomas. J Neurosurg 1991; 75(5):702-708.

(13) Murillo-Bonilla LM, Cantu-Brito C, rauz-Gongora A, Higuera-Calleja J, Padilla-Rubio J, Barinagarrementeria-Aldatz F. [Cavernous angioma. Clinical observations and prognosis of 133 patients]. [Spanish]. Rev Invest Clin 2003; 55(4):387-393.

(14) Cantu C, Murillo-Bonilla L, Arauz A, Higuera J, Padilla J, Barinagarrementeria F. Predictive factors for intracerebral hemorrhage in patients with cavernous angiomas. Neurol Res 2005; 27(3):314-318.

(15) Aiba T, Tanaka R, Koike T, Kameyama S, Takeda N, Komata T. Natural history of intracranial cavernous malformations. J Neurosurg 1995; 83(1):56-59.

(16) Kondziolka D, Lunsford LD, Kestle JR. The natural history of cerebral cavernous malformations. J Neurosurg 1995; 83(5):820-824.

(17) Kim DS, Park YG, Choi JU, Chung SS, Lee KC. An analysis of the natural history of cavernous malformations. Surg Neurol 1997; 48(1):9-17.

(18) Labauge P, Brunereau L, Laberge S, Houtteville JP. Prospective follow-up of 33 asymptomatic patients with familial cerebral cavernous malformations. Neurology 2001; 57(10):1825-1828.

(19) Kupersmith MJ, Kalish H, Epstein F, Yu G, Berenstein A, Woo H et al. Natural history of brainstem cavernous malformations. Neurosurgery 2001; 48(1):47-53.

(20) Labauge P, Brunereau L, Levy C, Laberge S, Houtteville JP. The natural history of familial cerebral cavernomas: a retrospective MRI study of 40 patients. Neuroradiology 2000; 42(5):327-332.

(21) Porter PJ, Willinsky RA, Harper W, Wallace MC. Cerebral cavernous malformations: natural history and prognosis after clinical deterioration with or without hemorrhage. J Neurosurg 1997; 87(2):190-197.

(22) Moriarity JL, Wetzel M, Clatterbuck RE, Javedan S, Sheppard JM, Hoenig-Rigamonti K et al. The natural history of cavernous malformations: a prospective study of 68 patients. Neurosurgery 1999; 44(6):1166-1171.

(23) Porter RW, Detwiler PW, Spetzler RF, Lawton MT, Baskin JJ, Derksen PT et al. Cavernous malformations of the brainstem: experience with 100 patients. J Neurosurg 1999; 90(1):50-58.

(24) Feiz-Erfan I, Zabramski JM, Lanzino G, Porter RW. Natural history of cavernous malformations of the brain. Operative Techniques in Neurosurgery 2002; 5(3):171-175.

(25) Ebrahimi A, Etemadifar M, Ardestani PM, Maghzi AH, Jaffe S, Nejadnik H. Cavernous angioma: a clinical study of 35 cases with review of the literature. Neurol Res 2009; 31(8):785-793.

(26) Abdulrauf SI, Kaynar MY, Awad IA. A comparison of the clinical profile of cavernous malformations with and without associated venous malformations. Neurosurgery 1999; 44(1):41-46.

(27) Menon G, Gopalakrishnan CV, Rao BRM, Nair S, Sudhir J, Sharma M. A single institution series of cavernomas of the brainstem. J Clin Neurosci 2011; 18:1210-1214.

(28) Barker II FG, Amin-Hanjani S, Butler WE, Lyons S, Ojemann RG, Chapman PH et al. Temporal clustering of hemorrhages from untreated cavernous malformations of the central nervous system. Neurosurgery 2001; 49(1):15-24.

(29) Hasegawa T, McInerney J, Kondziolka D, Lee JY, Flickinger JC, Lunsford LD. Long-term results after stereotactic radiosurgery for patients with cavernous malformations. Neurosurgery 2002; 50(6):1190-1197.

(30) Mathiesen T, Edner G, Kihlstrom L. Deep and brainstem cavernomas: A consecutive 8-year series. J Neurosurg 2003; 99(1):31-37.

(31) Wang C-C, Liu A, Zhang J-T, Sun B, Zhao Y-L. Surgical management of brain-stem cavernous malformations: Report of 137 cases. Surg Neurol 2003; 59(6):444-454.

(32) Ghannane H, Khalil T, Sakka L, Chazal J. [Analysis of a series of cavernomas of the central nervous system: 39 non operated cases, 39 operated cases, 1 dead]. [French]. Neuro-Chirurgie 2007; 53(2-3 Pt 2):217-222.
